# Nicotine Increases Macrophage Survival through α7nAChR/NF-κB Pathway in *Mycobacterium* *avium* *paratuberculosis* Infection

**DOI:** 10.3390/microorganisms9051086

**Published:** 2021-05-18

**Authors:** Dania AlQasrawi, Ebraheem Naser, Saleh A. Naser

**Affiliations:** 1 Division of Molecular Microbiology, Burnett School of Biomedical Sciences, College of Medicine, University of Central Florida, Orlando, FL 32816, USA; daniaqasrawi@Knights.ucf.edu; 2 College of Pharmacy, University of Florida, Gainesville, FL 32611, USA; ebraheemnaser@ufl.edu

**Keywords:** nicotine, MAP, macrophages, α7nAChR, NF-κB

## Abstract

Recently, we reported that nicotine plays a role in the failure of the macrophage in the clearance of *Mycobacterium avium* subspecies *paratuberculosis* (MAP) during infection in Crohn’s disease smokers. We also demonstrated that nicotine enhances macrophages cellular survival during MAP infection. Blocking α7 nicotinic acetylcholine receptor (α7nAChR) with the pharmacological antagonist—mecamylamine—subverted the anti-inflammatory effect of nicotine in macrophages. Yet, it is still unknown how α7nAChR is involved in the modulation of the macrophage response during MAP infection. Here, we studied the mechanistic role of nicotine-α7nAChR interaction in modulating NF-ĸB survival pathway, autophagy, and effect on cathelicidin production in MAP-infected macrophages using THP-1 cell lines. Our results showed that nicotine upregulated α7nAChR expression by 5-folds during MAP infection compared to controls. Bcl-2 expression was also significantly increased after nicotine exposure. Moreover, Nicotine inhibited autophagosome formation whereas infection with MAP in absence of nicotine has significantly increased LC-3b in macrophages. Nicotine also further upregulated NF-ĸB subunits expression including Rel-B and p100, and increased nuclear translocation of p52 protein. We also discovered that cathelicidin production was significantly suppressed in MAP-infected macrophages, treatment with nicotine showed no effect. Overall, the study provides new insight toward understanding the cellular role of nicotine through α7nAChR/NF-ĸB p100/p52 signaling pathway in inducing anti-apoptosis and macrophage survival during MAP infection in Crohn’s disease smokers.

## 1. Introduction

The cigarette smoke (CS) epidemic is a fatal public health problem resulting in over 8 million deaths per year [1]. The World Health Organization (WHO) reported that about 7 million of these deaths are due to the direct consumption of tobacco, while the remaining 1 million is the result of passive smoking [1]. It is widely accepted that CS exacerbates symptoms in Crohn’s disease (CD) patients [2]. Epidemiological studies have reported that tobacco consumption increases the risk of developing CD. Specifically, it has been reported that individuals who smoke more than 20 cigarettes a day have a two to four times increased risk of CD relapse [3]. Similarly, other studies have elucidated that smokers are five times more prone to develop active CD than non-smoking individuals [3]. Moreover, it has been reported that the risk of death is seven times higher for smokers than it is for never-smokers [3]. The WHO estimates that 73% of CD deaths worldwide are attributed to smoking [3]. On the other hand, bacterial infection caused by *Mycobacterium avium* subspecies *paratuberculosis* (MAP), adherent-invasive *Escherichia coli* (AIEC), and *Klebsiella pneumoniae* are strongly associated with CD, which is exacerbated by smoking [4,5,6]. However, the molecular mechanisms by which exposure to CS increases the susceptibility to bacterial infection in CD patients is not well understood.

Nicotine, an alkaloid found naturally in tobacco plants, is the most active agent in CS and thought to have the greatest effect on the human body [7]. Nicotine can directly pass the cell membranes in low concentrations without any interaction with a receptor due to its lipophilic characteristics [7]. However, the major effects of nicotine are mediated through receptors; nicotine mimics acetylcholine and acts as an agonist to the nicotinic acetylcholine receptors (nAChRs), which are present in presynaptic neurons, postsynaptic neurons, and muscle cells. Nicotinic receptors are also present in immune cells such as monocytes and macrophages [8]. The nAChRs are transmembrane proteins composed of five subunits arranged around a central cation-selective pore [9]. Neuronal nAChRs can exist as homooligomeric proteins, comprised only of α subunits, or as heterooligomeric proteins, composed of a combination of α and β subunits [10]. So far, ten α subunits and four β subunits have been identified [11]. Among all nAChRs, α7 nicotinic receptor has been described as the most widely expressed receptor in macrophages. Nicotine-induced activation of α7 in macrophages stimulates the cholinergic anti-inflammatory pathway by inhibiting the production of pro-inflammatory cytokines such as interleukin (IL)-6, tumor necrosis factor alpha (TNF-α), and IL-8, which are necessary for macrophages’ defense against microbes [11]. We previously reported that preventing nicotine binding to its receptor, α7nAChR, using a pharmacologic antagonist significantly attenuated the anti-inflammatory effect of nicotine and partially abrogated the ability of macrophages to maintain MAP viability [12]. These findings indicate that nicotine binding to α7nAChR is an important mechanism by which nicotine impairs the macrophage response against MAP.

Nicotine also has the ability to inhibit cellular apoptosis and autophagy with subsequent increased bacterial burden in macrophages [12]. This may be considered another mechanism by which nicotine impairs macrophage effector function against MAP. Although there are reports of the effects of nicotine on key molecules involved in the macrophage response against MAP, it is still not known how α7nAChR is involved in the modulation of the macrophage response during MAP infection [3,12]. The present study aims to investigate the mechanism by which nicotine inhibits cellular apoptosis and possible involvement of α7nAChR in the activation of NF-ĸB caused by nicotine using an in-vitro MAP infection model with a THP-1 cell line.

## 2. Materials and Methods

### 2.1. Monocyte-Derived Macrophages Preparation and Cell Culture

The THP-1 monocytes (ATCC TIB-202) were cultured in RPMI-1640 medium (ATCC 30-2001) containing 10% fetal bovine serum (FBS; Sigma Life Science, St. Louis, MO, USA). The cells then were maintained in a humidified 5% CO-2 incubator at 37 °C to reach 80% confluency. About 1 × 10^5^ cells were transferred to 12-well tissue culture plates with. Then, the cells were stimulated into monocyte-derived macrophages using 50 ng/mL phorbol 12-myristate 13-acetate (PMA; Sigma Life Science, St. Louis, MO, USA) followed by 24 h of incubation. All experiments were performed in duplicates and done within eight passages.

In some experiments, monocyte-derived macrophages were infected with 1 × 10^7^ CFU/mL of MAP strain UCF4 (a clinical strain isolated from CD patient), *M. tuberculosis* ATCC HR237, or stimulated with 5 µg/mL lipopolysaccharide (LPS) from *E. coli* strain O111.B4 (Sigma Life Science, St. Louis, MO, USA) incubated at 37 °C for 24 h, followed by nicotine treatment (10^−7^−10^−8^ M) for additional 24 h of incubation at the same conditions.

### 2.2. Measurement of Gene Expression in Treated Macrophages

*RNA Isolation*: To isolate RNA, treated cells were centrifuged at 2500 rpm for 5 min at 4 °C and then cell pellets were suspended in 500 µL of TRIzol^®^ reagent (Invitrogen, Carlsbad, CA, USA). A 125 µL of chloroform was added to each sample, mixed and incubated at room temperature 5 min. Next, the samples were centrifuged at 10,000 rpm for 5 min and the top aqueous phase was collected and transferred to a new 2.0 mL microcentrifuge tube. These were then gently mixed with 275 µL isopropanol. Following centrifugation at 14,000 rpm and 4 °C for 20 min, RNA pellets were washed in 500 µL of 75% ethanol by centrifugation at 9500 rpm for 5 min at 4 °C. After that, ethanol was poured off and the samples were air-dried for 10 min, and then dissolved in 15 µL of Tris-EDTA (TE) buffer. RNA concentrations were measured using NanoDrop (OD at 260 nm).

*cDNA Synthesis:* cDNA synthesis was carried out as described earlier [13]. Briefly, 800 ng of RNA was added to microtubes containing 20 µL of PCR reaction; 4 µL iScript™ Reverse transcription (Bio-Rad^®^) and up to 20 µL RNAse-free water. The reaction was performed using MyGene Series Peltier Thermal Cycler under the following conditions: 5 min at 25 °C, 20 min at 46 °C and 1 min at 95 °C. The cDNA samples were either stored at −20 °C or used immediately for RT-qPCR analysis.

*RT-PCR:* gene expression analysis was performed as described earlier [14]. For each sample, a 1 µL of cDNA (30 ng/µL) was mixed with10 µL of Fast SYBR Green Mastermix (Thermo Fisher Scientific, Waltham, MA, USA), 1 µL of forward primer, 1 µL of reverse primer of either *cholinergic receptor nicotinic alpha 7 subunit (CHRNA7), REL-B, Nuclear Factor kappa B subunit 2 (p100).*

*(NFκB2), Bcl-2, LC-3b, or Cathelicidin* (Thermo Fisher Scientific, Waltham, MA, USA) (Table 1), and up to 20 µL of RNAse-free water, were prepared in a 96-well Microamp RT-PCR reaction plate and placed analyzed using 7500 Fast Real-Time PCR System (Applied Biosystems, Foster City, CA, USA). Housekeeping GAPDH primer was used to obtain baseline CT readings. Relative mRNA expression as fold change was calculated by using the equation 2^(−∆∆CT)^, where $\Delta \mathrm{CT}={\mathrm{CT}}_{\mathrm{sample}}-{\mathrm{CT}}_{\mathrm{GAPDH}}$ whereas ∆∆ CT = ∆ CT_Treated_−∆ CT_Untreated_.

### 2.3. Measurement of Cathelicidin Protein Expression in Treated Macrophages

Following 24 h of nicotine treatment, the infected macrophages were pelleted by centrifugation at 2500 rpm for 5 min at 4 °C. Next, the pellets were incubated with iced RIPA buffer (Thermo-Fisher, Waltham, MA, USA) for 20 min, then centrifuged at 14,000 rpm for 20 min at 4 °C. Supernatant containing cell lysates were analyzed using ELISA kits: Human Cathelicidin High Sensitivity ELISA, which was purchased from LifeSpan BioSciences (Seattle, WA, USA). All groups were tested in triplicate.

### 2.4. Preparation of Macrophages Nuclear Protein Extract

Nuclei were extracted from 1.0 mL of each infected macrophages suspension after 24 h of nicotine treatment, using the nuclear and cytoplasmic extraction kit by MyBioSource (San Diego, CA, USA) according to the manufacturer’s protocol. Briefly, cells were centrifuged at 500× *g* for 5 min and then washed with cold 1X PBS. Next, cells were suspended in 500 μL of 5X Complete Cytoplasmic Extraction Buffer. Following 5 min of incubation on ice, the samples were centrifuged at 3000× *g* for 4 min at 4 °C. After transferring out the supernatant, the nuclear pellets were washed with 1–2 mL of Nuclear Wash Buffer and then mixed with 2X Complete Nuclear Extraction Buffer containing protease inhibitor cocktail (PIC). Samples were then incubated on ice for 30 min on a shaking platform at 4 °C. Finally, suspensions were centrifuged at 13,500× *g* for 10 min at 4 °C and the supernatants containing nuclear fractions were saved at −80 °C until further use. Protein concentrations were quantified using NanoDrop (OD at 280 nm).

### 2.5. Measurement of NF-κB p100/p52 Transcription Factor Activation in Treated Macrophages

The NF-κB p100/p52 (Phospho-Ser865) Colorimetric DNA-Binding ELISA Kit by TFact^TM^ (San Diego, CA, USA) was used to determine NF-κB activation according to the manufacturer’s protocol. Briefly, the samples containing nuclear extracts were added to wells, which were coated with a dsDNA sequence specific to NF-κB p100/p52, and the plate then was incubated for 2 h at RT. The plate was then washed 3 times using 1X wash buffer to remove unbound reagents. NF-κB p100/p52 primary antibody was added to each well and the plate was incubated for 1 h on an orbital shaker at RT. Following three times washes, diluted transcription factor goat anti-rabbit HRP conjugate secondary antibody was added and incubated for an additional 1 h on an orbital shaker at RT. Following three washes, developing reagents were added to the wells for 10–30 min until blue color development. When color development is sufficient, Stop solution was added to each well and absorbance was read at 450 nm. Percent activation scores were calculated by the equation: (Sample absorbance reading/positive control absorbance reading) ∗ 100.

### 2.6. Statistical Analysis

GraphPad Prism V.7.02 (GraphPad, La Jolla, CA, USA) was performed to analyze data statistics. Significance among experiments was assessed by Unpaired Two-tailed t test at *p* < 0.05 and a 95% confidence interval (CI). All data collected in this study were pre-tested for normal distribution using the Kolmogorov–Smirnov normality test. Data is shown as (Mean ± SD). *p*-values < 0.001 is also mentioned when achieved.

## 3. Results

### 3.1. Nicotine Significantly Upregulates Nicotinic Acetylcholine Receptor (α7nAChR) Expression in MAP-Infected Macrophages

**Figure 1:**
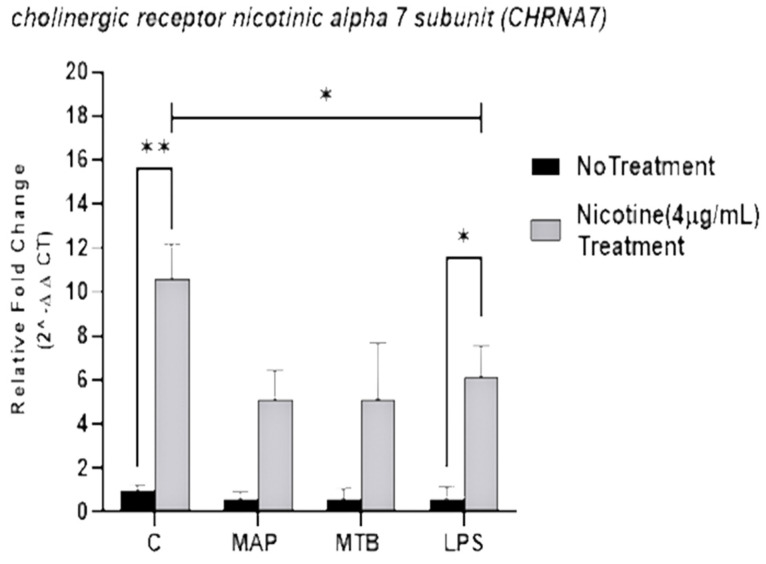
Effect of nicotine on the *α7nAChR* expression on infected macrophages. THP-1 PMA differentiated macrophages were infected with MAP and MTB, or treated with LPS for 24 h, then treated with nicotine (4 µg/mL). Expression of *α7nAChR* was measured by RT-PCR. All experiments were performed in duplicate. MAP: *Mycobacterium avium paratuberculosis*. MTB: *Mycobacteria tubercalusis*. LPS: lipopolysaccharide derived from *Escherichia coli* ATCC 8739. * *p* < 0.05, ** *p* < 0.001.

The effect of nicotine was examined on α7nAChR expression on macrophages, following infection with MAP or MTB infection or treatment with LPS treatment for 24 h. Our results indicate that nicotine significantly elevated *α7nAChR* expression on uninfected macrophages (10.57 ± 1.61; *p* < 0.001) while macrophages with no nicotine treatment exhibited the lowest levels of *α7nAChR* (0.48 ± 0.22); these groups were used as reference controls. It is worth mentioning that the effect of MAP infection on *α7nAChR* was not significant when compared with uninfected untreated cells (Figure 1). However, there was an increase in *α7nAChR* expression after nicotine treatment in MAP-infected macrophages (MAP + nicotine: 5.12 ± 1.32 vs. MAP alone: 0.62 ± 0.31; *p* < 0.05). Similar trend was also observed in MTB-infected macrophages. Additionally, nicotine caused significant upregulation of *α7nAChR* expression in macrophages treated with LPS comparing with macrophages treated with LPS and not with nicotine (LPS + nicotine: 6.15 ± 1.10 vs. LPS alone: 20.45 ± 2.4; *p* < 0.05).

### 3.2. Nicotine Induces Modulation of Anti-Apoptotic Gene (Bcl2) in MAP-Infected Macrophages

**Figure 2:**
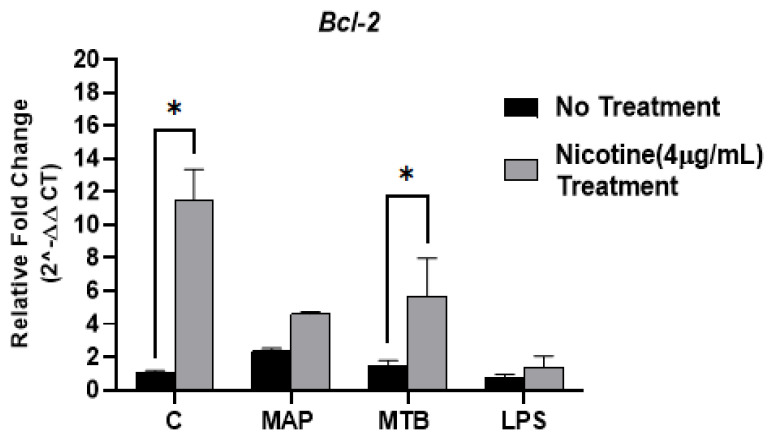
Effect of nicotine on the *Bcl2* expression in infected macrophages. THP-1 PMA differentiated macrophages were infected with MAP and MTB, or treated with LPS for 24 h, then treated with nicotine (4 µg/mL). Expression of *Bcl2* was measured by RT-PCR. All experiments were performed in duplicate. MAP: *Mycobacterium avium paratuberculosis*. MTB: *Mycobacteria tubercalusis*. LPS: lipopolysaccharide derived from *Escherichia coli* ATCC 8739. * *p* < 0.05.

*Bcl2* expression analysis showed that nicotine treatment substantially increased the expression of this regulator in macrophages (11.50 ± 1.85; *p* < 0.05) compared to macrophages with no nicotine treatment (1.01 ± 0.15; *p* < 0.05). Infection with MAP or MTB did not exhibit significant changes in *Bcl2* (MAP: 2.33 ± 0.18, MTB: 1.50 ± 0.29; *p* < 0.05; Figure 2) while adding nicotine treatment following mycobacterial infection elevated its expression (MAP + nicotine: 4.60 ± 0.11, MTB + nicotine: 5.71 ± 2.26; *p* < 0.05). Interestingly, there was no clear change in *Bcl2* expression when macrophages treated with LPS with or without nicotine treatment.

### 3.3. Nicotine Decreases Autophagy Marker Expression (LC-3b) While MAP Significantly Increases It

**Figure 3:**
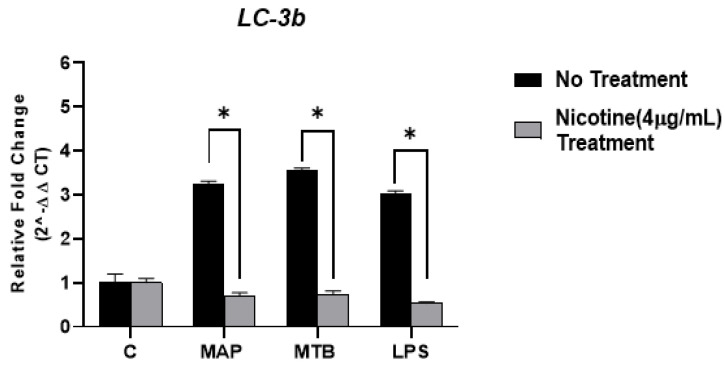
Effect of nicotine on the *LC-3b* expression in infected macrophages. THP-1 PMA differentiated macrophages were infected with MAP and MTB, or treated with LPS for 24 h, then treated with nicotine (4 µg/mL). Expression of *LC-3b* was measured by RT-PCR. All experiments were performed in duplicate. MAP: *Mycobacterium avium paratuberculosis*. MTB: *Mycobacteria tubercalusis*. LPS: lipopolysaccharide derived from *Escherichia coli* ATCC 8739. * *p* < 0.05.

To assess whether nicotine also affects autophagy marker (LC-3b) in macrophages, the gene expression of this marker was measured using RT-PCR after infection and/or nicotine stimulation. MAP-infected macrophages with no nicotine treatment exhibited high level of *LC-3b* (3.25 ± 0.06; *p* < 0.05). In parallel to these results, similar findings were observed in MTB-infected macrophage or LPS-treated macrophages and the expression of *LC-3b* was highest (3.56 ± 0.04 and 3.02 ± 0.07; *p* < 0.05, respectively) compared to uninfected macrophages (1.01 ± 0.18; *p* < 0.05). In contrast, nicotine significantly reduced *LC-3b* expression in mycobacterial infected macrophages (MAP: 0.69 ± 0.08, MTB: 0.74 ± 0.07; *p* < 0.05; Figure 3). Likewise, *LC-3b* expression was also lowered in LPS-treated macrophages in response to nicotine treatment (0.53 ± 0.03; *p* < 0.05).

### 3.4. Nicotine Upregulates the Expression of REL-B and NF-ĸB p100 Subunit in MAP-Infected Macrophages

**Figure 4:**
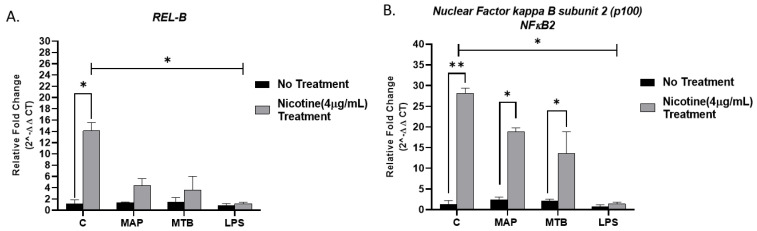
Effect of nicotine on the (**A**) *REL-B* and (**B**) *NF-ĸB p100* subunit expressions in infected macrophages. THP-1 PMA differentiated macrophages were infected with MAP and MTB, or treated with LPS for 24 h, then treated with nicotine (4 µg/mL). Expression of *REL-B* and *NF-ĸB p100* were measured by RT-PCR. All experiments were performed in duplicate. MAP: *Mycobacterium avium paratuberculosis*. MTB: *Mycobacteria tubercalusis*. LPS: lipopolysaccharide derived from *Escherichia coli* ATCC 8739. * *p* < 0.05, ** *p* < 0.001.

We evaluated the expression of *REL-B* and *NF-ĸB p100* subunit in MAP or MTB-infected macrophages and LPS-treated macrophages in response to nicotine treatment using RT-PCR-based approach. Nicotine significantly increased *REL-B* and *NF-ĸB p100* expressions in uninfected macrophages (14.09 ± 1.47 and 28.18 ± 0.07; *p* < 0.05; Figure 4) compared to macrophages with no nicotine treatment; these groups were used as reference controls. *REL-B* and *NF-ĸB p100* expressions in MAP-infected macrophages with no nicotine treatment were 1.34 ± 0.12 and 2.51 ± 0.52. Relative to these MAP findings, nicotine showed significant upregulation of *REL-B* and *NF-ĸB p100* expressions with 4.34 ± 1.25 and 18.9 ± 0.89, respectively. Similarly, nicotine significantly elevated *REL-B* and *NF-ĸB p100* expressions in MTB-infected macrophages. Additionally, there were an increase in the expression of these genes in LPS-treated macrophages in response to nicotine stimulation but in less pattern (1.15 ± 0.26 and 1.4 ± 0.41; *p* < 0.05, respectively).

### 3.5. Nicotine Increases p52 Protein and NF-ĸB (Survival Pathway) Activity in MAP-Infected Macrophages

NF-κB p52 activation was determined in MAP, MTB-infected macrophages and LPS-treated macrophages treated with nicotine (Table 2). Activation scores represent the proportion of activated NF-κB p52, as indicated by nuclear translocation. In addition, fold changes was calculated in comparison to our reference control. Our results indicated that nicotine treatment significantly increased NF-κB p52 activation in uninfected macrophages by 2.44 folds compared with our reference control (86.78% vs. 35.44%). Nicotine also elevated NF-κB p52 activation in MAP-infected macrophages (65.20%) and LPS-treated macrophages (58.41%). However, the greatest elevation in NF-κB p52 activation was observed in MTB-infected macrophages with nicotine treatment compared to other treated infected macrophages, *p* < 0.001. Interestingly, there was a reduction in NF-κB p52 activation that was even less than the reference control when the macrophages were treated with LPS, although not with nicotine (23.70%).

### 3.6. Nicotine Synergistically Inhibits Cathelicidin Production in MAP-Infected Macrophages

**Figure 5:**
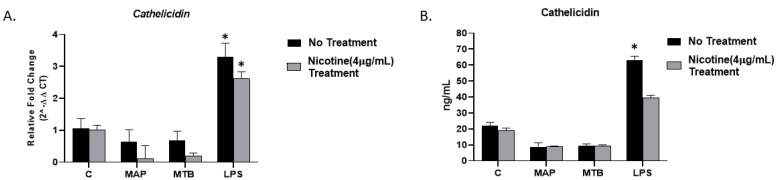
Effect of nicotine in cathelicidin expression on infected macrophages. THP-1 PMA differentiated macrophages were infected with MAP and MTB, or treated with LPS for 24 h, then treated with nicotine (4 µg/mL). Expression of cathelicidin was measured by (**A**) RT-PCR and (**B**) ELISA, respectively. All experiments were performed in duplicates. MAP: *Mycobacterium avium paratuberculosis*. MTB: *Mycobacteria tubercalusis*. LPS: lipopolysaccharide derived from *Escherichia coli* ATCC 8739. * *p* < 0.05.

Previous studies showed that nicotine inhibits the production of antimicrobial peptide; cathelicidin [15]. Here we studied a possible role of nicotine in impairing macrophages immunity against MAP infection by suppressing production of cathelicidin. Our results indicated that the synergy of MAP and nicotine exhibited the lowest expression of *cathelicidin* (0.11 ± 0.41; *p* < 0.05, Figure 5A) compared to all other groups. Likewise, a reduction in *cathelicidin* expression was also observed in MTB-infected macrophages in response to nicotine treatment (0.20 ± 0.09; *p* < 0.05, Figure 5A). Surprisingly, there was a significant elevation in *cathelicidin* expression in LPS-treated macrophages regardless nicotine stimulation. The findings were consistent with results from protein level analysis (Figure 5B) (MAP + nicotine: 8.94 ± 0.35 vs. MAP alone: 8.67 ± 2.61; *p* < 0.05), (MTB + nicotine: 9.37 ± 0.62 vs. MTB alone: 9.63 ± 1.10; *p* < 0.05), or (LPS + nicotine: 39.38 ± 1.74 vs. LPS alone: 63.03 ± 2.4; *p* < 0.05).

## 4. Discussion

Although nicotine is the most active agent in cigarette smoke, which is responsible for addiction, it is also considered to have the least harmful effects on human health compared to other tobacco components [16]. Previous studies have demonstrated that the anti-inflammatory effects of nicotine may offset other harmful response induced by cigarette smoke in many clinical conditions such as Ulcerative Colitis, Parkinson’s disease and Alzheimer’s disease [16]. On the other hand, other studies have demonstrated that α7nAChR is the major subunit that binds to nicotine on macrophages, which suggests that α7nAChR may be involved in a regulatory mechanism of the inflammatory response in macrophages [17,18]. These findings were consistent with our previous report, which revealed that blocking α7nAChR with a pharmacological antagonist subverted the anti-inflammatory effect of nicotine in macrophages [12]. On the other hand, nicotine binding to α7nAChR abrogated the macrophages immunity against MAP, which is considered the most studied microbial causative agent of CD [12,19,20,21]. However, little is known about the mechanism involved in how α7nAChR mediated nicotine affects infected macrophages. In this regard, the present study concerning to assay the expression levels of α7nAChR, NF-ĸB subunits, Rel-B, Bcl2 and Cathelicidin in infected macrophages, to confirm whether they were involved in this process.

Although nicotine is lipophilic and capable of passing through cells passively, our results showed a significant increase in the expression of α7nAChR in infected macrophages after nicotine treatment (Figure 1). These findings are not surprising given the absence of nicotine in reference to control has been shown low expression of α7nAChR which may clearly support a possible non-synaptic role of α7nAChR in macrophages regardless of bacterial infection. Previous studies have been demonstrated that α7nAChR is an important component of the cholinergic anti-inflammatory pathway [22]. Additionally, other studies have revealed that nicotine has a more pronounced effect than acetylcholine on activation of the cholinergic anti-inflammatory pathway via α7nAChR [23]. Taken together, we suggest that nicotine causes an alteration in immune responses in bacterial infected macrophages by α7nAChR -mediated mechanism.

Since autophagy and apoptosis are the most efficient killing mechanisms of macrophages against intracellular mycobacteria, we previously reported that nicotine inhibits the classical apoptosis by decreasing the caspase-3 activity and maintains MAP viability in macrophages [12]. Moreover, other studies suggested that nicotine decreases induction of apoptosis and autophagy in alveolar macrophages infected with MTB [24]. Therefore, in this study, we continued our investigation about the effect of nicotine on other antiapoptotic regulators and autophagic markers including Bcl2 and LC-3b in macrophages infected with MAP, as a CD-associated pathogen. Consequently, our data showed a significant elevation in the expression of Bcl2 in macrophages after nicotine stimulation while MAP infection alone did not alter the expression of Bcl2 (Figure 2). Clearly, nicotine has an important role in promoting cellular survival in macrophages regardless of bacterial infection by increasing the expression of Bcl2. On the other hand, our findings confirmed that mycobacterial infection, specifically MAP, induces the autophagy process, an effective killing mechanism for macrophages, while nicotine impairs macrophage immunity against MAP by reducing the expression of autophagic marker, LC-3b (Figure 3). Hence, this modulation in macrophage survival regulators could be related to the susceptibility of diseases related to tobacco consumption and bacterial infection, such as CD.

This led us to investigate the pathway involved in α7nAChR-mediated activation of survival genes in infected macrophages while under nicotine stress. Specifically, we characterized the effect of nicotine in non-canonical NF-ĸB pathway in macrophages with MAP infection. Previous studies have indicated the involvement of NF-ĸB pathway in tobacco smoke-induced anti-apoptotic proteins [25]. In present study, nicotine significantly enhanced the expression of non-canonical NF-ĸB subunits, specifically Rel-B and p100 in MAP-infected macrophages (Figure 4). Nicotine also increased NF-ĸB p52 translocation and activation in macrophages in presence or absence of MAP infection (Table 2). This suggests that nicotine activates the antiapoptotic effect of NF-ĸB in MAP-infected macrophages, which may induce several antiapoptotic genes including inhibit caspase-3 activation [12,26]. These results support previous studies that reported that inhibiting NF-ĸB in macrophages leads to increased apoptosis and reduced viability of intracellular MTB [24]. Another study in vivo has shown that NF-ĸB activation in mouse macrophages increased killing of non-pathogenic *Mycobacterium smegmatis* [27,28]. These studies combined with our findings, support the role of nicotine in NF-ĸB activation in macrophages with mycobacterial infection while the antiapoptotic effect of NF-ĸB in macrophages depends on the host species and the microbial pathogen.

An interesting finding in these results was that MAP infection has a modest decrease in the level of cathelicidin; an innate antimicrobial component in macrophages, while macrophages treated with LPS significantly increased it. These data supported our previous report that indicated involvement of TLR2 in MAP infection since TLR2 ligands strongly downregulate cathelicidin expression in macrophages [27,29]. A subsequent study revealed that vitamin D counteracted downregulation of cathelicidin by *M. tuberculosis.* Taken together, we suggest that MAP uses inhibition of cathelicidin expression as a strategy to evade antimicrobial mechanisms in macrophages (Figure 5). To our knowledge, this is the first report showing gene expression and production of cathelicidin in human macrophages after MAP infection or LPS treatment. Paradoxically, nicotine has no effect in cathelicidin level in macrophage in presence or absence of bacterial infection.

**Figure 6:**
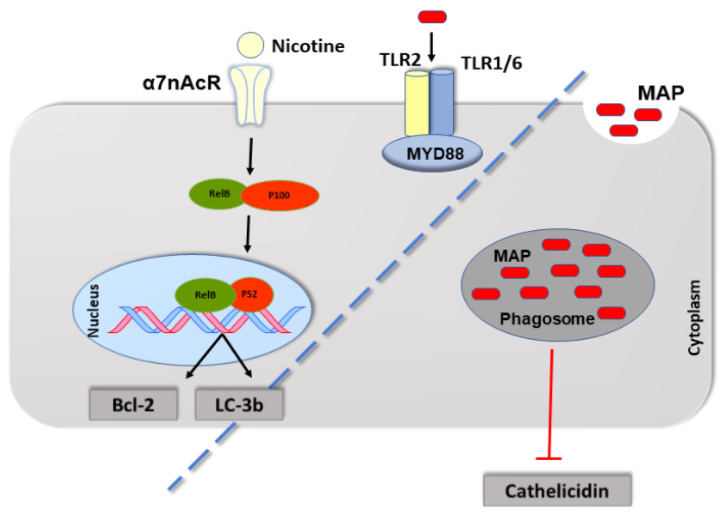
Schematic illustration of the role of nicotine in activation NF-ĸB survival pathway in MAP-infected macrophages. Nicotine through its receptor, α7nAChR, upregulates Bcl-2 and LC-3b expressions in MAP-infected macrophages. Meanwhile MAP itself evades macrophages antimicrobial defense by inhibiting cathelicidin.

In summary, we showed that nicotine could induce antiapoptotic gene expression mainly through α7nAChR subunit, which activates the NF-ĸB p100/p52 signaling pathway in MAP-infected macrophages (Figure 6). These findings may add new understanding on the potential pharmacological effects of α7nAChR in the treatment of inflammatory diseases with bacterial infection. Further research is also needed to validate the beneficial role of vitamin D in cathelicidin induction for treatment of CD with MAP infection.

## Figures and Tables

**Table 1:** RT-PCR primer sequences for tested genes.

Gene	Forward Primer Sequence (5′→3′)	Reverse Primer Sequence (5′→3′)
*GAPDH*	5′-CTTTTGCAGACCACAGTCCATG-3′	5′-TTTTCTAGACGGCAGGTCAGG-3′
*CHRNA7*	5′-GCTCGTCACGTGGAGAGG-3′	5′-GGGAGGCAGTGGCTTTACC-3′
*REL-B*	5′-CGCGATCGTCCACCAGA-3′	5′-AGACTACTCCGGGACCGC-3′
*NFκB2*	5′-GCCCACCCCCATTTAGATCTG-3′	5′-TGAGAGCATCTGCGAGCATAC-3′
*Bcl-2*	5′-CGCGATCGTCCACCAGA-3′	5′-AGACTACTCCGGGACCGC-3′
*LC-3b*	5′-CCACAGCTAGCAGCTGAACT-3′	5′-GGCTGTCTGGTGATTCCTGTAAA-3′
*Cathelicidin*	5′-AGGGATGGGTGGATCAGGAA-3′	5′-CGAAGCACAGCTTCCTTGTAG-3′

**Table 2:** Activation Score of Phosphorylated NF-κB p100/p52 (%).

Treatment Group	Phosphorylated NF-κB Activity Score ± SD (%)	Fold Change in Phosphorylated NF-κB Activity
Control	35.44 ± 1.07	-
Nicotine (4 µg/mL)	68.78 ± 6.1	2.44
MAP	44.45 ± 2.7	1.25
MAP + Nicotine (4 µg/mL)	65.20 ± 10.8	1.89
MTB	41.29 ± 4.4	1.16
MTB + Nicotine (4 µg/mL)	73.12 ± 23.3	2.06
LPS	23.70 ± 2.4	0.66
LPS + Nicotine (4 µg/mL)	58.41 ± 12.1	1.60

N: nicotine. MAP: *Mycobacterium avium paratuberculosis*. MTB: *Mycobacterium tuberculosis*. LPS: lipopolysaccharide derived from *Escherichia coli* ATCC 8739.

## Data Availability

Not Applicable.

## References

[1] Valdez-Miramontes C.E., Martínez L.A.T., Torres-Juárez F., Carlos A.R., Marin-Luévano S.P., de Haro-Acosta J.P., Enciso-Moreno J.A., Rivas-Santiago B. (2020). Nicotine modulates molecules of the innate immune response in epithelial cells and macrophages during infection with *M. tuberculosis*. Clin. Exp. Immunol..

[2] Thomas G.A.O., Rhodes J., Ingram J.R. (2005). Mechanisms of disease: Nicotine—A review of its actions in the context of gastrointestinal disease. Nature Clin. Pract. Gastroenterol. Hepatol..

[3] AlQasrawi D., Qasem A., Naser S.A. (2020). Divergent Effect of Cigarette Smoke on Innate Immunity in Inflammatory Bowel Disease: A Nicotine-Infection Interaction. Int. J. Mol. Sci..

[4] Naser S.A., Sagramsingh S.R., Naser A.S., Thanigachalam S. (2014). Mycobacterium avium subspecies paratuberculosis causes Crohn’s disease in some inflammatory bowel disease patients. World J. Gastroenterol. WJG.

[5] Rashid T., Wilson C., Ebringer A. (2013). The link between ankylosing spondylitis, Crohn’s disease, Klebsiella, and starch consumption. Clin. Dev. Immunol..

[6] Nazareth N., Magro F., Machado E., Ribeiro T.G., Martinho A., Rodrigues P., Alves R., Macedo G.N., Gracio D., Coelho R. (2015). Prevalence of Mycobacteriumavium subsp. paratuberculosis and Escherichia coli in blood samples from patients with inflammatory bowel disease. Med Microbiol. Immunol..

[7] Benfer B.A. (1997). Source Book of Substance Abuse and Addiction. J. Psychosoc. Nurs. Ment. Health Serv..

[8] Kazuto M., Klein T.W., Friedman H., Yamamoto Y. (2001). Involvement of nicotinic acetylcholine receptors in suppression of antimicrobial activity and cytokine responses of alveolar macrophages to Legionella pneumophila infection by nicotine. J. Immunol..

[9] Karlin A. (2002). Emerging structure of the nicotinic acetylcholine receptors. Nature Rev. Neurosci..

[10] Báez-Pagán C.A., Delgado-Vélez M., Lasalde-Dominicci J.A. (2015). Activation of the macrophage α7 nicotinic acetylcholine receptor and control of inflammation. J. Neuroimmune Pharmacol..

[11] Wang H., Yu M., Ochani M., Amella C.A., Tanovic M., Susarla S., Li J.H., Wang H., Yang H., Ulloa L. (2003). Nicotinic acetylcholine receptor α7 subunit is an essential regulator of inflammation. Nature.

[12] Dania A., Abdelli L.S., Naser S.A. (2020). Mystery Solved: Why Smoke Extract Worsens Disease in Smokers with Crohn’s Disease and Not Ulcerative Colitis? Gut MAP!. Microorganisms.

[13] Qasem A., Naser S.A. (2018). TNFα inhibitors exacerbate Mycobacterium paratuberculosis infection in tissue culture: A rationale for poor response of patients with Crohn’s disease to current approved therapy. BMJ Open Gastroenterol..

[14] Qasem A., Elkamel E., Naser S.A. (2020). Anti-MAP Triple Therapy Supports Immunomodulatory Therapeutic Response in Crohn’s Disease through Downregulation of NF-κB Activation in the Absence of MAP Detection. Biomedicines.

[15] Radek K.A., Elias P.M., Taupenot L., Mahata S.K., O’Connor D.T., Gallo R.L. (2010). Neuroendocrine nicotinic receptor activation increases susceptibility to bacterial infections by suppressing antimicrobial peptide production. Cell Host Microbe.

[16] Li Q., Zhou X.-D., Kolosov V.P., Perelman J.M. (2011). Nicotine reduces TNF-α expression through a α7 nAChR/MyD88/NF-ĸB pathway in HBE16 airway epithelial cells. Cell. Physiol. Biochem..

[17] Gwilt C.R., Donnelly L.E., Rogers D.F. (2007). The non-neuronal cholinergic system in the airways: An unappreciated regulatory role in pulmonary inflammation?. Pharmacol. Ther..

[18] Chong I.-W., Lin S.-R., Hwang J.-J., Huang M.-S., Wang T.-H., Hung J.-Y., Paulauskis J.D. (2002). Expression and regulation of the macrophage inflammatory protein-1alpha gene by nicotine in rat alveolar macrophages. Eur. Cytokine Netw..

[19] Qasem A., Abdel-Aty A., Abu-Suwa H., Naser A.S. (2016). Oxidative stress due to Mycobacterium avium subspecies paratuberculosis (MAP) infection upregulates selenium-dependent GPx activity. Gut Pathog..

[20] Qasem A., Naser A.E., Naser S.A. (2017). The alternate effects of anti-TNFα therapeutics and their role in mycobacterial granulomatous infection in Crohn’s disease. Expert Rev. Anti-Infect. Ther..

[21] Qasem A., Ramesh S., Naser S.A. (2019). Genetic polymorphisms in tumour necrosis factor receptors (TNFRSF1A/1B) illustrate differential treatment response to TNFα inhibitors in patients with Crohn’s disease. BMJ Open Gastroenterol..

[22] Park S.-Y., Baik Y.H., Cho J.H., Kim S., Lee K.-S., Han J.-S. (2008). Inhibition of lipopolysaccharide-induced nitric oxide synthesis by nicotine through S6K1-p42/44 MAPK pathway and STAT3 (Ser 727) phosphorylation in Raw 264.7 cells. Cytokine.

[23] Takahashi H.K., Iwagaki H., Hamano R., Kanke T., Liu K., Sadamori H., Yagi T., Yoshino T., Tanaka N., Nishibori M. (2007). The immunosuppressive effects of nicotine during human mixed lymphocyte reaction. Eur. J. Pharmacol..

[24] Bai X., Feldman N.E., Chmura K., Ovrutsky A.R., Su W.-L., Griffin L., Pyeon D., McGibney M.T., Strand M.J., Numata M. (2013). Inhibition of nuclear factor-kappa B activation decreases survival of Mycobacterium tuberculosis in human macrophages. PLoS ONE.

[25] Lee H.H., Dadgostar H., Cheng Q., Shu J., Cheng G. (1999). NF-κB-mediated up-regulation of Bcl-x and Bfl-1/A1 is required for CD40 survival signaling in B lymphocytes. Proc. Natl. Acad. Sci. USA.

[26] AlQasrawi D., Naser S.A. (2020). Nicotine Modulates MyD88-Dependent Signaling Pathway in Macrophages during Mycobacterial Infection. Microorganisms.

[27] Gutierrez M.G., Mishra B.B., Jordao L., Elliott E., Anes E., Griffiths G. (2008). NF-κB activation controls phagolysosome fusion-mediated killing of mycobacteria by macrophages. J. Immunol..

[28] Gutierrez M.G., Gonzalez A.P., Anes E., Griffiths G. (2009). Role of lipids in killing mycobacteria by macrophages: Evidence for NF-κB-dependent and-independent killing induced by different lipids. Cell. Microbiol..

[29] Rode A.K.O., Kongsbak M., Hansen M.M., Lopez D.V., Levring T.B., Woetmann A., Ødum N., Bonefeld C.M., Geisler C. (2017). Vitamin D counteracts Mycobacterium tuberculosis-induced cathelicidin downregulation in dendritic cells and allows Th1 differentiation and IFNγ secretion. Front. Immunol..

